# The complete mitochondrial genome of *Hemathlophorus brevigenatus* Wei, 2005 (Hymenoptera: Tenthredinidae) with phylogenetic analysis

**DOI:** 10.1080/23802359.2021.1967806

**Published:** 2021-08-25

**Authors:** Jiafen Liu, Meicai Wei, Gengyun Niu

**Affiliations:** College of Life Sciences, Jiangxi Normal University, Nanchang, China

**Keywords:** Allantini, *Hemathlophorus brevigenatus*, mitochondrial genome, phylogenetic analysis

## Abstract

The mitochondrial genome of *Hemathlophorus brevigenatus* Wei, 2005 collected from Huanggang Mountain of China is described using the NGS approach. The length of the sequence is 15,452 bp containing 13 protein-coding genes, 22 transfer RNA genes, two ribosomal RNA genes, and one control region. The overall A + T content is 79.5%. tRNA rearrangements occur in the MQI cluster. Phylogenetic analysis of *H. brevigenatus* resolved it in a clade with *Allantus togatus* in Allantinae which provides new evidence for the phylogeny of Tenthredinidae.

Lacourt (1996) erected the tribe Athlophorini for *Hemathlophorus* Malaise, 1945 and *Athlophorus* Burmeister, 1847 under the subfamily Sioblinae of Tenthredinidae. Although *Hemathlophorus* and *Athlophorus* are two peculiar genera among the Tenthredinidae as shown by the fore wing with a long vein R + M and a more or less basally constricted abdomen, Lacourt’s peculiar systematic arrangement of the genera and the tribe was not adopted by sawfly researchers, such as Wei and Nie (1998), Saini (2006), and Taeger et al. (2010). In the three later systems, *Hemathlophorus* and *Athlophorus* were placed into the Allantinae of Tenthredinidae. In this study, we sequenced the mitochondrial genome of *Hemathlophorus brevigenatus* Wei, 2005 and inferred its phylogenetic history with other mitochondrial genomes of Symphytan species to clarify the phylogenetic position of *Hemathlophorus* within the Tenthredinidae.

Specimens of *H. brevigenatus* were collected from Huanggangshan Mountain, Fujian Province, China (27.81 N, 117.95 E) in April 2019. The specimen was deposited at the Asia Sawfly Museum, Nanchang (ASMN) (Meicai Wei, weimc@126.com) under the voucher number CSCS-Hym-MC0177. Whole genomic DNA was extracted from the thorax muscle of a female adult using the DNeasy Blood &Tissue Kit (Qiagen, Valencia, CA). Genomic DNA was analyzed with the high-throughput Illumina Hiseq 4000 platform with 150 bp paired-end reads. DNA sequences were assembled using MitoZ (Meng et al. 2019) and Geneious Prime 2019.2.1 (Biomatters Ltd., Auckland, New Zealand). A total of 43,205,384 raw reads were assembled using MitoZ and resulted in a contig 15,108 bp in length with 37 genes. The control region was assembled using *trnM, trnI* from the *Xenapatidea procincta* (MW487928) as a reference. The contig was extended 3′ by 117 bp and 5′ by 582 bp using the “Map to Reference” function in Geneious Prime. This extension that resulted overlapped with the 15,108 bp contig and closed the gap. Thus, a 482 bp long control region was obtained. The verification is conducted by assembly using *X. procincta* as the reference. The annotations of tRNAs were generated using the MITOS web server (Bernt et al. 2013). Protein-coding genes (PCGs) were annotated by the open reading frames between the flanking tRNAs, and then defined by comparative analyses (Cameron 2014).

The complete mitochondrial genome sequences were aligned with ClustalW using default settings (Thompson et al. 1994) and concatenated with SequenceMatrix v1.7.8 (Vaidya et al. 2011). The phylogenetic tree was constructed using IQ-TREE (maximum-likelihood) (Nguyen et al. 2015) with the GTR + MTART model and 1000 bootstrap replicates. To investigate the phylogenetic position of *H. brevigenatus*, 10 unsaturated nucleotide sequences (*atp8, nad2*, *nad6* are excluded) of 37 Hymenopterans are aligned separately then concatenated, resulting in an alignment of 9836 bp.

The total length of the complete mitochondrial genome is 15,452 bp, containing 13 PCGs, 22 transfer RNA genes, two ribosomal RNA genes, and one control region. The mitochondrial base composition is A 42.5%, T 37.0%, G 7.8%, and C 12.7%. The AT content is 79.5% and is common in the Tenthredinidae (Ma et al. 2019). All of the PCGs are initiated with the ATN (ATT, ATA, and ATG) codon, except *nad4l* which initiated from GTG. Among these genes, three PCGs (*nad1*, *nad5*, and *nad6*) initiated with ATA, seven PCGs (*atp6*, *cob*, *cox1*, *cox2*, *cox3*, *nad2*, and *nad4*) with ATG and two PCGs (*atp8* and *nad3*) initiated from ATT. All the PCGs have a TAA termination codon except *nad4* which has an incomplete terminal codon T—. Compared with the ancestral insect (*Drosophila*) mitochondrial genome (Boore 1999), the control region*- trnI (+)- trnQ (-)- trnM (+)* is rearranged to *trnM (-)- trnQ (+)-* control region*- trnI (+)* in *H. brevigenatus*. There are five gene overlapping regions that appeared among *trnI-nad2* (1 bp), *trnW-trnC* (1 bp), *atp6*-*atp8* (7 bp), *atp6*-*cox3* (1 bp), and *nad6*-*cob* (1 bp). The mitogenome has nine intergenic spacers with a total length of 173 bp in 18 locations varying in size from 1 to 39 bp with the longest located between *trnF* and *nad5*.

Phylogenetic analysis of *H. brevigenatus* fully resolved it in a clade with *Allantus togatus* (MW464859) and *Allantoides luctifer* (KJ713152) classified in the Allantinae as suggested by Wei and Nie (1998) and Taeger et al. (2010). Its placement was remote from the *Siobla* of Tenthredininae and clearly denied Lacourt’s system (Lacourt 1996). The phylogenetic relationships of Tenthredininae are inferred as (*Athalia* + ((*Conaspidia* + Selandriinae) ((Hoplocampinae + Nematinae) ((Tenthredininae + Allantinae) + ((Fenusinae + Blennocampinae) + Caliroinae))))) (Figure 1). All related files are publicly available in figshare (https://figshare.com/account/home#/projects/114354).

**Figure 1.**
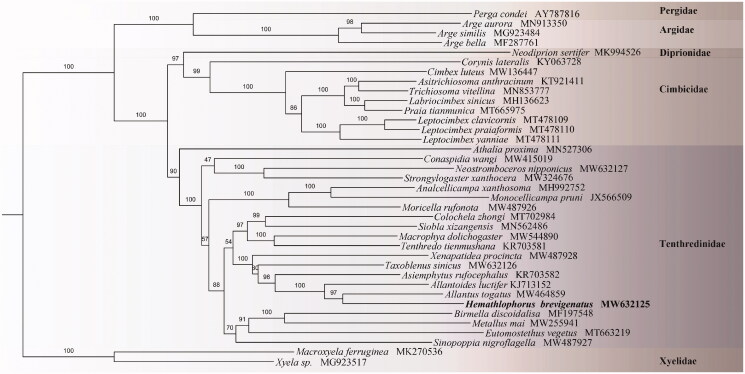
Phylogenetic tree including *Hemathlophorus brevigenatus* based on the combination of 10 unsaturated nucleotide sequences. Numbers on each node correspond to the bootstrap support values based on 1000 replicates.

## Data Availability

The genome sequence data that support the findings of this study are openly available in GenBank of NCBI at [https://www.ncbi.nlm.nih.gov] (https://www.ncbi.nlm.nih.gov/) under the accession number MW632125. The associated BioProject, SRA, and BioSample numbers are PRJNA714776, SRR14233978, and SAMN18397095 respectively.
